# Decoding the effects of Bexarotene treatment on brain of AD‐like model mice: single‐cell transcriptomics and chromatin accessibility analysis

**DOI:** 10.1002/alz70855_103001

**Published:** 2025-12-23

**Authors:** Yi Lu, Xuebao Wang, Nicholas F Fitz, Radosveta Koldamova, Iliya Lefterov

**Affiliations:** ^1^ University of Pittsburgh, Pittsburgh, PA, USA

## Abstract

**Background:**

Bexarotene, an RXR‐specific agonist, has shown neuroprotective effects in Alzheimer's disease (AD) mouse models by improving cognition and increasing amyloid‐beta (Aβ) clearance. RXR activation regulates gene networks involved in neural development, neuroinflammation, and metabolism. This study aimed to examine how Bexarotene alters chromatin architecture and gene activity in the brains of APP/PS1 AD mice.

**Methods:**

APP/PS1de9 mice were treated with Bexarotene (100 mg/kg/d for 10 days) or vehicle (corn oil/DMSO). Mouse brains were dissociated, and cDNA libraries were generated using the 10X platform for single‐cell sequencing. Dimensionality reduction and unsupervised clustering were performed with the Seurat pipeline to identify cell populations and analyze gene expression. Changes in chromatin architecture were examined with single‐cell ATAC‐seq (snATAC‐seq). Data from both scRNA‐seq and snATAC‐seq were integrated using the ArchR pipeline, and differentially accessible peaks were identified using the Signac package. Transcription factor (TF) activity was analyzed with TOBIAS and TF‐COMB, with ChIP‐seq used for validation.

**Results:**

We present a single‐cell‐resolution map of both transcriptomic and epigenomic changes in Bexarotene‐treated AD model mice. We profiled the transcriptional and epigenomic landscapes of 37,640 cells (scRNA‐seq) and 61,353 nuclei (snATAC‐seq) from 8 samples (4 Bexarotene‐ and 4 vehicle‐treated controls). We identified clusters corresponding to astrocytes, microglia, oligodendrocytes, neurons, blood vessel cells, and macrophages. Gene expression analysis revealed transcriptional responses to Bexarotene, with many genes involved in developmental processes and lipid metabolism pathways. Integration of snATAC‐seq and scRNA‐seq identified cell‐type‐specific TFs, and differentially accessible chromatin regions. Microglia and endothelial cells exhibited the most significant changes, with genes primarily associated with developmental functions. TF footprinting and cooccurrence analysis further revealed the binding activity and regulatory network of RXR and its partnered heterodimers, providing insights into the transcriptional regulation underlying the observed gene expression changes in response to Bexarotene treatment.

**Conclusions:**

Bexarotene upregulates RXR‐controlled gene networks in major brain cell types, including microglia and endothelial cells. The combined analysis of single‐cell transcriptomics, ATAC‐seq, and ChIP‐seq provides new insights into how RXR activation may restore brain homeostasis by regulating neuroinflammation, amyloid deposition, and neuronal damage. This research highlights the complexity of TF signaling pathways and their potential for therapeutic use in AD.

